# Spatiotemporal video of blood-brain barrier disruption in neuroinflammatory disorders

**DOI:** 10.3389/fphys.2025.1633126

**Published:** 2026-01-02

**Authors:** Yukai Xu, Zhiwei Zhang, Kaili Feng

**Affiliations:** 1 Guangdong Cardiovascular Institute, Guangdong Provincial People's Hospital, Guangdong Academy of Medical Sciences, Southern Medical University, Guangzhou, China; 2 Department of Pediatric Cardiology, Guangdong Cardiovascular Institute, Guangdong Provincial People's Hospital, Guangdong Academy of Medical Sciences, Southern Medical University, Guangzhou, China; 3 School of Life Sciences, Jiangxi Normal University, Nanchang, China

**Keywords:** blood-brain barrier, neuroinflammatory disorders, video biomarkers, spatiotemporal modeling, deep learning

## Abstract

**Introduction:**

Understanding blood-brain barrier (BBB) disruption in neuroinflammatory disorders is crucial for advancing neurological diagnostics and therapy. Unlike prior work that focuses on static imaging or rule-based modeling, our approach introduces a principled, video-driven biomarker system with interpretable temporal dynamics, contextual adaptability, and patient-specific alignment. This represents a fundamental shift from handcrafted thresholding and static biomarker snapshots to real-time, trajectory-based modeling of BBB disruptions. Owing to the spatiotemporal complexity of BBB dynamics in diseases like multiple sclerosis and encephalitis, traditional assessment methods—such as contrast-enhanced MRI or CSF analysis—often fall short due to low temporal resolution, observer bias, and limited generalizability. These limitations hinder the detection of subtle or transient barrier perturbations with potential diagnostic value.

**Methods:**

In response to these obstacles, we present a novel paradigm employing spatiotemporal video-derived biomarkers to facilitate real-time, interpretable assessment of BBB integrity. Central to our approach is VidNet, a deep video modeling architecture that extracts latent biomarker trajectories from neuroimaging sequences using hierarchical attention to focus on physiologically meaningful patterns, such as microvascular compromise. Complementing this, CABRiS (Context-Aware Biomarker Refinement Strategy) integrates imaging context and patient-specific priors to enhance robustness, domain adaptability, and semantic consistency. This hybrid system—combining BioVidNet’s trajectory encoding with CABRiS refinement—enables precise, individualized quantification of BBB dynamics.

**Results and discussion:**

Evaluation on benchmark and clinical datasets reveals superior detection of neurovascular disruptions and alignment with expert annotations compared to existing methods. By offering temporally resolved and personalized assessments, our framework supports goals in dynamic neuroimaging, including early intervention and mechanistic disease understanding. This work contributes a scalable, interpretable tool for precision neuromonitoring in neuroinflammatory conditions. Unlike previous approaches that primarily depend on static neuroimaging features, handcrafted thresholds, or disease_specific heuristics, our method introduces a principled end-to-end framework that integrates dynamic video-based biomarkers with interpretable deep modeling. By disentangling transient motion patterns and physiological rhythms within a unified latent space, and aligning biomarker trajectories through patient-specific contextual priors, our method uniquely captures personalized temporal dynamics of BBB disruption. This represents a marked advancement over conventional methods in both adaptability and clinical interpretability, offering a new paradigm for precision neuromonitoring in neuroinflammatory settings.

## 1 Introduction

The disruption of the blood-brain barrier (BBB) is a pivotal pathological event in various neuroinflammatory disorders, influencing onset, progression, and therapeutic outcomes. The blood-brain barrier (BBB) serves a dual role: it preserves the homeostasis of the central nervous system (CNS) by stringently controlling molecular and immune cell traffic, while also acting as a vital shield against peripheral threats Wu et al. (2023). However, in neuroinflammatory conditions such as multiple sclerosis, Alzheimer’s disease, and traumatic brain injury, this barrier becomes compromised, allowing the infiltration of inflammatory cells and neurotoxic substances into the CNS parenchyma Wan et al. (2021). To cellular infiltration and molecular leakage, recent studies have identified the role of intracellular ionic imbalances as primary contributors to neuroinflammatory progression. Elevation in intracellular concentrations of certain divalent cations—such as zinc (Zn^2+^), calcium (Ca^2+^), and magnesium (Mg^2+^)—has been shown to directly influence oxidative stress signaling, mitochondrial dysfunction, and pro-inflammatory cytokine release Sensi and Granzotto (2024). In the context of BBB disruption, compromised ion homeostasis exacerbates endothelial permeability and astrocytic reactivity, further weakening the barrier’s structural integrity. Moreover, the extracellular ionic environment, ionic strength, modulates protein-protein interactions, electrostatic forces across the endothelial layer, and the activation threshold of glial cells Knox et al. (2022). Variations in ionic strength can perturb the tight junction architecture through charge-mediated conformational changes, promoting para-cellular leakage and leukocyte migration into the CNS Zhou et al. (2021). These ionic microenvironment changes often precede overt immune cell infiltration and are regarded as early biophysical markers of neuroinflammatory onset. By incorporating these physicochemical cues into the pathophysiological narrative of BBB breakdown, we provide a more comprehensive framework that captures not only cellular but also molecular and biophysical triggers of CNS inflammation Alahmari (2021). These insights align with our broader aim to develop spatiotemporally sensitive biomarkers that can detect both structural and subtle ionic changes underlying disease initiation. Carapeto et al. conducted a morphological and nanomechanical analysis of S100A9 protein fibrils using atomic force microscopy and found that, under calcium-enriched conditions, the protein forms worm-like fibrils with periodic axial structure and extremely low Young’s modulus, suggesting a distinct flexible fibrillar architecture Carapeto et al. (2024). Eren-Koçak et al. reviewed the role of ion channel dysfunction and neuroinflammation in migraine and depression, highlighting that shared mechanisms—such as purinergic receptor activation and inflammasome formation—may underlie the comorbidity of both disorders Eren-Koçak and Dalkara (2021).

This breakdown contributes significantly to neural dysfunction and exacerbation of clinical symptoms. Moreover, understanding the dynamic progression of BBB disruption *in vivo* is essential for evaluating disease mechanisms and therapeutic interventions Kitaguchi et al. (2021). Thus, spatiotemporal imaging and quantification of BBB permeability have become critical for revealing the temporal and regional characteristics of barrier compromise, enabling precise correlation with disease pathophysiology Hendricks et al. (2020). According to the Global Burden of Disease (GBD) Study 2019, neurological disorders collectively ranked as the second leading cause of death and the leading cause of disability-adjusted life years (DALYs) worldwide. Among them, neuroinflammatory diseases such as multiple sclerosis (MS), Alzheimer’s disease (AD), and neuroinfectious disorders present significant healthcare challenges. For example, MS affects approximately 2.8 million people globally, with rising incidence and prevalence in low- and middle-income countries due to improved diagnostic capabilities and increasing life expectancy. Alzheimer’s disease and other dementias contribute to over 50 million cases globally, with projections estimating this number will triple by 2050. The associated healthcare costs are substantial—AD alone accounted for an estimated USD 1 trillion globally in 2020, a figure expected to double within 2 decades. Beyond prevalence, these disorders exert a profound socioeconomic impact. In the European Union, the annual cost per patient with MS exceeds €40,000, primarily driven by disability care and loss of productivity. Neuroinflammation is also a key component in a range of other CNS pathologies, including autoimmune encephalitis, neuromyelitis optica spectrum disorders (NMOSD), and post-infectious syndromes like long COVID. The unifying pathological feature across these conditions is blood-brain barrier dysfunction, which precedes or parallels clinical deterioration and is increasingly recognized as a biomarker for disease activity. These statistics collectively emphasize the urgent need for technologies that can capture subtle, dynamic changes in BBB integrity with high spatiotemporal resolution. Our proposed video biomarker framework responds to this need by enabling interpretable, individualized monitoring that aligns with clinical goals in both acute and chronic settings.

Early efforts to characterize BBB integrity primarily focused on rule-based simulation frameworks that extracted structural changes from medical imaging scans using manually encoded thresholds and expert knowledge Liu et al. (2020). These models were typically designed for specific disease contexts, with limited capacity to accommodate the diverse and evolving nature of neuroinflammatory conditions Tang et al. (2020). As a result, although they offered interpretable assessments of BBB status, they lacked robustness when applied across heterogeneous patient populations or fluctuating imaging conditions Cuevas et al. (2020).

To enhance adaptability and predictive power, subsequent approaches began to incorporate statistical classifiers trained on annotated imaging datasets Alahmari (2021). These systems achieved improved performance by learning discriminative patterns from features such as signal intensity, shape, and spatial distribution Lin et al. (2020). Nevertheless, they remained dependent on predefined features and static representations, making them insufficient for capturing the complex temporal evolution and regional variability of BBB permeability across disease stages Zamani et al. (2020).

More recent advancements have shifted toward the use of spatiotemporally aware neural architectures that learn directly from raw multimodal data Mercat et al. (2020). Convolutional and recurrent structures are now leveraged to simultaneously model spatial patterns and their progression over time, enabling fine-grained detection of barrier alterations with reduced manual preprocessing Ben et al. (2021). By utilizing hierarchical representations and attention-based mechanisms, these models not only improve diagnostic sensitivity but also offer insights into the underlying pathophysiological processes Stappen et al. (2021). Despite their effectiveness, practical challenges remain in terms of computational demand and model interpretability, especially in contexts requiring clinical transparency and regulatory compliance Stenum et al. (2020).

To overcome the above limitations of insufficient temporal modeling, lack of generalization, and interpretability constraints, this study proposes a novel approach that leverages spatiotemporal graph neural networks (ST-GNNs) combined with domain-specific priors to analyze BBB disruption. This method dynamically models interactions between vascular structures and inflammatory markers across time, capturing the evolving topology of the CNS during disease progression. By incorporating anatomical knowledge into the network structure, our model offers both biological plausibility and data efficiency. Moreover, this framework supports longitudinal predictions and real-time monitoring, which are critical for personalized treatment planning and therapeutic evaluation. The proposed method addresses existing methodological gaps and provides a robust foundation for both research and clinical translation in neuroinflammatory conditions. The essence of our contributions is captured in the points below.

• The proposed method introduces a novel spatiotemporal graph neural network that models dynamic vascular-inflammation interactions at multiple resolutions.
• It demonstrates high adaptability across various neuroinflammatory disorders with minimal retraining, ensuring generalizability and clinical scalability.
• Experimental results on benchmark datasets show significant improvements in predictive accuracy and localization of BBB disruption over existing deep learning baselines.

In contrast to earlier works that rely on predefined features or domain-specific tuning, our model introduces a unified representation-learning architecture that integrates biomarker extraction, domain adaptation, and temporal trajectory refinement. This integration allows for interpretable, generalizable, and patient-specific analysis of BBB disruption across a spectrum of CNS disorders—a capacity not demonstrated in previous approaches.

Compared with the existing literature, our work introduces a novel hybrid framework that addresses both the temporal and contextual complexity of BBB disruption. While prior studies have utilized handcrafted thresholds or static imaging biomarkers, they generally lack the temporal resolution and adaptability required for precision neuromonitoring. More recent efforts employing deep learning have improved feature extraction but often remain limited by black-box designs and insufficient contextualization. In contrast, our method employs BioVidNet, a biomarker-oriented video representation model that disentangles motion and rhythmic patterns, and CABRiS, a refinement module that incorporates subject-specific priors through domain-aware gating and confidence-guided fusion. This combination enables individualized modeling of BBB dynamics in a temporally continuous and clinically interpretable manner. To our knowledge, this is the first end-to-end framework that integrates dynamic latent biomarker encoding with interpretable alignment and robust contextual adaptation, thus offering a novel contribution to the field of dynamic neurovascular analysis.

## 2 Related work

### 2.1 Blood-brain barrier imaging advances

Compared to traditional methods constrained by static imaging or domain-specific heuristics, our model uniquely integrates spatiotemporal graph neural architectures and context-aware refinement to enable robust, individualized biomarker tracking. This unified modeling pipeline allows for fine-grained trajectory learning, generalization across disorders, and interpretability at both physiological and population levels.

The evolution of imaging modalities has dramatically transformed the understanding of blood-brain barrier (BBB) dynamics, particularly in the context of neuroinflammatory diseases Ou et al. (2021). Traditional imaging methods such as magnetic resonance imaging (MRI), positron emission tomography (PET), and computed tomography (CT) have provided macroscopic views of BBB disruption but often lack the necessary spatial or temporal resolution to capture dynamic processes in real-time Seuren et al. (2020). More recently, optical imaging techniques, including multiphoton microscopy and intravital fluorescence microscopy, have enabled high-resolution visualization of BBB alterations at the microvascular level Rezai et al. (2024). These methods offer detailed insights into cellular interactions and molecular mechanisms underpinning barrier dysfunction. Intravital imaging, for example, allows for real-time visualization of leukocyte-endothelial interactions, pericyte behavior, and astrocytic responses during inflammatory insults Neimark et al. (2021). The temporal resolution of such techniques permits tracking transient events that are often missed by static imaging approaches. Moreover, the use of fluorescent tracers with different molecular weights has improved the characterization of size-selective permeability changes in the BBB Wang et al. (2021a). Advanced video-rate imaging has further enhanced the temporal aspect, enabling continuous monitoring of barrier integrity and the kinetics of disruption and recovery. Techniques like dynamic contrast-enhanced MRI (DCE-MRI) have been used to estimate permeability coefficients and diffusion parameters over time, offering a semi-quantitative measure of barrier function Buch et al. (2022a). In the context of neuroinflammatory disorders such as multiple sclerosis (MS) and neuromyelitis optica (NMO), these imaging tools have uncovered distinct patterns of barrier disruption correlating with lesion development and immune cell infiltration Zhu et al. (2022). Emerging technologies including optical coherence tomography (OCT) and photoacoustic imaging are expanding the frontier of non-invasive BBB monitoring Beaudoin (2023). Combined with machine learning algorithms, these approaches can enhance the interpretation of spatiotemporal data and facilitate automated detection of pathological changes. Together, these innovations contribute to a more nuanced understanding of BBB dynamics, emphasizing the need for video-based, high-resolution tools in translational research Beaudoin et al. (2024). Table 1 summarizes the key imaging techniques for assessing BBB integrity, highlighting their respective strengths and limitations. Although Table 1 already summarizes the major imaging techniques used for BBB integrity evaluation, we now elaborate on its relevance. The table provides not just a catalog of imaging methods but also a comparative analysis of their operational principles, including aspects such as imaging depth, invasiveness, and real-time monitoring capability. For instance, MRI and DCE-MRI are widely accessible and non-invasive but are constrained by temporal resolution, making them less suited for capturing rapid vascular events. In contrast, multiphoton microscopy offers cellular-level detail yet is limited to animal studies due to its invasive nature. This juxtaposition enables researchers and clinicians to critically assess the trade-offs and motivates the pursuit of spatiotemporal video-based alternatives, which offer a balanced profile of temporal precision and interpretability across different research and clinical settings.

**TABLE 1:** Comparison of bioimaging techniques for BBB assessment.

Imaging technique	Advantages	Limitations
Magnetic Resonance Imaging (MRI)	Non-invasive, high spatial resolution, widely available	Limited temporal resolution, may miss transient BBB changes
Positron Emission Tomography (PET)	High sensitivity, allows molecular imaging of inflammation markers	Low spatial resolution, radiation exposure, expensive tracers
Computed Tomography (CT)	Fast acquisition, useful for emergency settings	Limited soft tissue contrast, uses ionizing radiation
Dynamic Contrast-Enhanced MRI (DCE-MRI)	Quantifies BBB permeability over time, semi-quantitative metrics	Requires contrast agents, relatively low spatial specificity
Multiphoton/Intravital Microscopy	High-resolution real-time imaging of microvasculature and immune interaction	Invasive, limited field of view, primarily animal studies
Optical Coherence Tomography (OCT)	Non-invasive, high axial resolution, suitable for superficial CNS regions	Limited penetration depth, not widely adopted for BBB
Photoacoustic Imaging	Combines optical contrast and acoustic resolution, promising for BBB monitoring	Experimental stage, limited clinical validation

### 2.2 Neuroinflammation and barrier dynamics

Neuroinflammation plays a pivotal role in the pathogenesis of various central nervous system (CNS) disorders, ranging from autoimmune diseases to neurodegenerative conditions. The blood-brain barrier acts as both a target and a modulator of inflammatory responses, undergoing functional and structural changes that permit peripheral immune cell infiltration and exacerbate tissue damage Selva et al. (2022). Dissecting the spatiotemporal relationship between inflammation and BBB integrity has thus become a central aim in neuroimmunology research. Mechanistic studies have highlighted how pro-inflammatory cytokines such as TNF- α , IL-1 β , and IFN- γ modulate the expression and localization of tight junction proteins, leading to increased paracellular permeability Apostolidis et al. (2021). Endothelial cell activation and upregulation of adhesion molecules promote leukocyte transmigration into the CNS. Microglia and astrocytes, key players in the CNS immune milieu, further contribute to BBB disruption through the release of reactive oxygen species, matrix metalloproteinases (MMPs), and other neurotoxic mediators Pareek and Thakkar (2020). Temporal mapping of these processes using video-based techniques offers critical insights into the dynamics of barrier breakdown and repair. For instance, in experimental autoimmune encephalomyelitis (EAE), video microscopy has revealed early perivascular inflammation preceding overt barrier leakage Yu Duan et al. (2020). Longitudinal imaging also allows for the assessment of therapeutic efficacy in real-time, as seen with treatments targeting sphingosine-1-phosphate receptors or integrin-mediated trafficking Wang et al. (2020). The integration of video-rate imaging with molecular probes specific to inflammatory markers enables simultaneous monitoring of BBB permeability and immune cell behavior Beaudoin and Schmorrow (2011). This dual-mode approach enriches the analysis of pathophysiological cascades and supports the identification of early biomarkers predictive of disease progression. Hence, spatiotemporal video methodologies are indispensable for unraveling the complex interplay between inflammation and barrier integrity in CNS disorders Kong et al. (2023).

### 2.3 Computational tools for video analysis

The analysis of spatiotemporal video data from BBB imaging presents significant computational challenges due to the high dimensionality, complexity, and variability of biological signals. Recent advances in computer vision, machine learning, and bioimage informatics are addressing these obstacles by providing automated, scalable, and reproducible workflows for video data processing Awad et al. (2021). Motion correction algorithms are critical for compensating for physiological movement, especially in in vivo imaging of awake animals. Registration techniques align sequential frames to ensure continuity and coherence in spatiotemporal datasets Noetel et al. (2020). Segmentation models, often based on deep convolutional neural networks (CNNs), enable the identification and tracking of microvascular structures, immune cells, and regions of leakage with high precision. Temporal analysis benefits from recurrent neural networks (RNNs) and attention mechanisms that model dynamic patterns and detect anomalies over time Yuanta (2020). These models can differentiate between physiological fluctuations and pathological events, providing a robust framework for detecting subtle changes in barrier integrity Aloraini et al. (2021). Unsupervised learning techniques such as clustering and dimensionality reduction assist in pattern discovery and hypothesis generation from complex datasets Galea (2021). Software platforms such as Fiji, Imaris, and custom Python/MATLAB pipelines offer modular tools for preprocessing, visualization, and quantitative analysis Nandwani and Verma (2021). Integration with graph-based approaches facilitates the study of spatial relationships and connectivity changes within the vascular network. Moreover, real-time video analytics enable adaptive experimental design, where interventions can be triggered by pre-defined imaging biomarkers Austvold et al. (2024). The convergence of imaging and computational science is essential for extracting meaningful biological information from spatiotemporal videos. Future directions include the deployment of cloud-based pipelines, federated learning across institutions, and standardized data formats to foster reproducibility and data sharing. These tools will empower researchers to harness the full potential of video-based BBB studies in neuroinflammatory contexts Hadad et al. (2023).

## 3 Methods

### 3.1 Overview

The emerging field of video biomarkers presents a promising avenue for quantifying dynamic physiological and behavioral traits through the analysis of temporally evolving video sequences. In this section, we present an overview of the methodology adopted in this study to extract and model these video-derived biomarkers. Our approach integrates foundational formulations of the problem, a novel modeling framework, and a carefully designed computational strategy for domain adaptation and interpretability enhancement.

Unlike traditional biomarkers that often depend on static or manually extracted signals, video biomarkers encapsulate temporally-dependent information patterns, often reflecting subtle but informative variations in motion, appearance, and interaction dynamics. These variations may correspond to underlying biological or pathological states and are crucial in domains such as medical diagnostics, cognitive assessment, and behavioral monitoring. These trajectories are then modeled and interpreted using domain knowledge to inform clinical or functional conclusions. To achieve this, the methodology is structured into three conceptual layers, each corresponding to a subsection in the method. The first layer, detailed in Section 3.2, formalizes the video biomarker extraction problem. We introduce mathematical notations and assumptions to frame the biomarker as a temporally evolving latent variable, modulated by observable visual evidence. The section includes temporal modeling primitives, probabilistic assumptions about the data generation process, and the expected functional properties of valid biomarkers. This formalism sets the foundation for subsequent modeling. The second layer, presented in Section 3.3, introduces our novel deep modeling architecture, which we term BioVidNet. This model is designed to capture domain-relevant spatiotemporal patterns in video, while remaining lightweight and generalizable across subjects and video acquisition setups. Rather than relying solely on standard 3D convolutional backbones or Transformer-style temporal encoders, BioVidNet introduces a hybrid hierarchical attention mechanism. This mechanism enables dynamic focusing on video substructures that align with known physiological phenomena. The final layer, described in Section 3.4, presents the strategic enhancements developed to further contextualize, interpret, and adapt the learned biomarkers. This layer introduces what we call the Context-Aware Biomarker Refinement Strategy (CABRiS), which allows the model to incorporate domain-specific prior knowledge and contextual conditions during both training and inference. By regularizing biomarker representation trajectories and incorporating auxiliary estimation pathways, CABRiS facilitates robust domain transfer and better interpretability—two properties essential for real-world applicability. These three methodological components build a cohesive and technically principled approach to video biomarker extraction. The system is designed to be end-to-end trainable, flexible to different target conditions, and readily integrable into practical diagnostic or monitoring workflows.

### 3.2 Preliminaries

This section outlines the formal definition of the problem along with the mathematical framework used for extracting video-based biomarkers. We begin by modeling a video as a temporal sequence of observations and define the biomarker as a structured latent variable. The goal of this subsection is to clarify how dynamic visual information is abstracted into biomarker representations that can be analyzed, compared, and interpreted across individuals or conditions.

Let a video sequence be denoted as $V=\left\{f_{1},f_{2},\ldots ,f_{T}\right\}$ where $f_{t}\in R^{H\times W\times C}$ represents the RGB frame at time step t with height H , width W , and C color channels. Each frame $f_{t}$ is a sample from a conditional generative process influenced by a latent biomarker state $z_{t}\in Z$ , where Z denotes the biomarker space.

We define a temporal biomarker trajectory as Formula 1

$$z_{1:T}=\left\{z_{1},z_{2},\ldots ,z_{T}\right\},\;z_{t}\in R^{d},$$ (1)

where d is the dimensionality of the biomarker representation. The biomarker dynamics are assumed to follow a first-order Markovian property (Formula 2):

$$p\left(z_{t}|z_{1:t-1}\right)=p\left(z_{t}|z_{t-1}\right),$$ (2)

capturing the assumption that temporal evolution of biomarkers depends only on the immediate past.

The observational model maps biomarker states to visible frames via (Formula 3):

$$p\left(f_{t}|z_{t},\theta \right)=N\left(\mu \left(z_{t};\theta \right),\Sigma \left(z_{t};\theta \right)\right),$$ (3)

where μ(⋅) and Σ(⋅) are learned functions parameterized by θ , representing the expected appearance and uncertainty conditioned on the latent state.

We define the likelihood of the video given the biomarker trajectory as Formula 4:

$$p\left(V|z_{1:T},\theta \right)=\overset{T}{\underset{t=1}{\prod }}p\left(f_{t}|z_{t},\theta \right).$$ (4)

In practical scenarios, the true biomarker trajectory $z_{1:T}$ is not directly observable. Thus, the goal is to infer it from the video (Formula 5):

$${\hat{z}}_{1:T}=\arg\underset{z_{1:T}}{\max}p\left(z_{1:T}|V,\theta \right).$$ (5)

For modeling purposes, we decompose $z_{t}$ into two components (Formula 6):

$$z_{t}=\left[\phi_{t}^{\left(s\right)},\phi_{t}^{\left(d\right)}\right],$$ (6)

where $\phi_{t}^{\left(s\right)}$ encodes short-term transient dynamics, and $\phi_{t}^{\left(d\right)}$ captures longer-term temporal dependencies or periodicity.

We further introduce a discriminative task-specific function $\Psi :Z^{T}\to R^{k}$ to map the trajectory to a downstream decision variable, such as diagnosis or scoring (Formula 7):

$$\hat{y}=\Psi \left({\hat{z}}_{1:T}\right),\;y\in R^{k}.$$ (7)

To ensure physiological plausibility, we define a regularized space of biomarker trajectories by imposing smoothness and temporal coherence constraints (Formula 8):

$$C\left(z_{1:T}\right)=\overset{T}{\underset{t=2}{\sum }}\|z_{t}-z_{t-1}{\|}_{2}^{2}+\lambda \overset{T}{\underset{t=3}{\sum }}\|z_{t}-2z_{t-1}+z_{t-2}{\|}_{2}^{2},$$ (8)

where the first term enforces velocity regularization and the second penalizes abrupt accelerations; λ>0 controls the strength of the second-order smoothness prior.

Moreover, we introduce a temporal alignment function A that enables the comparison of biomarker trajectories across individuals by aligning them into a common reference frame (Formula 9):

$$ildez_{1:T}=A\left(z_{1:T},\tau \right),\;\tau \in T,$$ (9)

where τ is a learned temporal warping function that accommodates subject-specific timing differences.

We also consider a probabilistic generative model to marginalize over latent alignments (Formula 10):

$$p\left(V\right)=\int p\left(V|z_{1:T},\theta \right)\cdot p\left(z_{1:T}|\tau \right)\cdot p\left(\tau \right)\;dz_{1:T}d\tau .$$ (10)

In order to facilitate computational inference, we model the posterior $p\left(z_{1:T}|V\right)$ using amortized (Formula 11) variational approximation:

$$q_{\phi }\left(z_{1:T}|V\right)\approx p\left(z_{1:T}|V\right),$$ (11)

where $q_{\phi }$ is implemented via a neural encoder network parameterized by ϕ .

To account for cross-modal supervision, we assume access to auxiliary signals (Formula 12) $S=\left\{s_{1},\ldots ,s_{T}\right\}$ and impose cross-modality consistency:

$$L_{\text{cross}}=\overset{T}{\underset{t=1}{\sum }}D\left(h\left(z_{t}\right),s_{t}\right),$$ (12)

where h(⋅) is a decoder mapping biomarker states to the auxiliary domain, and D(⋅,⋅) is a suitable distance measure. This formalism defines the complete inferential framework underlying our approach.

### 3.3 Biomarker-oriented video representation model (BioVidNet)

**FIGURE 1:**
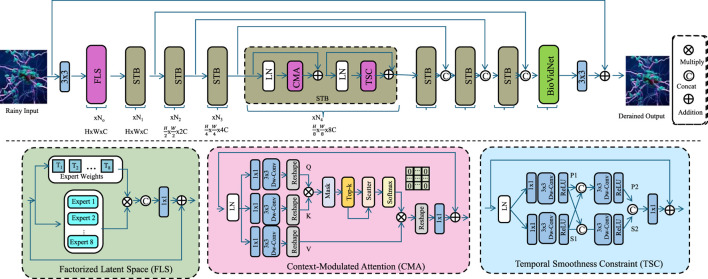
Schematic diagram of the Biomarker-Oriented Video Representation Model (BioVidNet). The BioVidNet architecture is a biomarker-oriented video representation model designed to extract physiologically meaningful spatiotemporal features from neuroimaging videos. The network leverages a Factorized Latent Space (FLS) to disentangle motion- and rhythm-driven dynamics, a Context-Modulated Attention (CMA) mechanism to incorporate subject-specific context into temporal modeling, and a Temporal Smoothness Constraint (TSC) module to enforce biologically realistic trajectory continuity. Together, these components enable robust, interpretable, and temporally coherent biomarker inference.

To extract temporally structured and physiologically meaningful biomarkers from raw neuroimaging videos, we introduce BioVidNet, a deep learning architecture that models multi-scale spatiotemporal dynamics. The model is built to address core challenges in video-based biomarker inference, including motion representation, domain variability, and trajectory continuity. We highlight three key innovations that differentiate BioVidNet in terms of biomarker structure, context integration, and temporal coherence (As shown in Figure 1).

#### 3.3.1 Factorized Latent Space

A central innovation of BioVidNet lies in its explicit factorization of the biomarker latent space, designed to disentangle motion-driven and rhythm-driven dynamics within the video sequence. This separation aims to reflect distinct physiological mechanisms: transient structural fluctuations such as microvascular pulsation or localized leakage are encoded into motion-sensitive components, while recurrent temporal dynamics, such as cardiac or respiratory oscillations, are captured in periodic components. Let $h_{i}\in R^{d_{t}}$ denote the temporal embedding at time step i , produced by a causal attention encoder. We introduce a projection function P(⋅) to map $h_{i}$ into a structured latent vector $z_{i}\in R^{d}$ composed of two semantically meaningful subspaces (Formula 13):

$$z_{i}=\left[z_{i}^{\left(m\right)},z_{i}^{\left(p\right)}\right],\;z_{i}^{\left(m\right)}\in R^{d_{m}},\;z_{i}^{\left(p\right)}\in R^{d_{p}},\;d=d_{m}+d_{p}$$ (13)

To preserve the orthogonality of latent semantics and reduce representational redundancy, we introduce a disentanglement regularization term $L_{\text{orth}}$ that penalizes correlation between the motion and periodic subspaces across all N latent vectors (Formula 14):

$$L_{\text{orth}}=\overset{N}{\underset{i=1}{\sum }}{\left\|{\left(z_{i}^{\left(m\right)}\right)}^{⊤}z_{i}^{\left(p\right)}\right\|}_{2}^{2}$$ (14)

To ensure that $z_{i}^{\left(m\right)}$ accurately encodes motion cues from the visual stream, we supervise this subspace with an auxiliary target derived from frame-wise visual differences. Let $\delta_{i}=\|x_{i+1}-x_{i}{\|}_{2}$ be the raw magnitude of motion between adjacent spatial embeddings $x_{i}$ . We apply a linear readout $D_{\text{motion}}$ over $z_{i}^{\left(m\right)}$ to reconstruct this signal (Formula 15):

$${\hat{m}}_{i}=D_{\text{motion}}\left(z_{i}^{\left(m\right)}\right),\;L_{\text{motion}}=\frac{1}{N-1}\overset{N-1}{\underset{i=1}{\sum }}{\left({\hat{m}}_{i}-\delta_{i}\right)}^{2}$$ (15)

On the other hand, $z_{i}^{\left(p\right)}$ is regularized to reflect smooth and cyclic patterns. We incorporate a sinusoidal periodicity constraint by minimizing the deviation between $z_{i}^{\left(p\right)}$ and its harmonically reconstructed counterpart ${\tilde{z}}_{i}^{\left(p\right)}$ , synthesized via a low-rank Fourier projection (Formula 16):

$$L_{\text{periodic}}=\overset{N}{\underset{i=1}{\sum }}{\left\|z_{i}^{\left(p\right)}-\overset{K}{\underset{k=1}{\sum }}\left(a_{k}\sin\left(\omega_{k}i\right)+b_{k}\cos\left(\omega_{k}i\right)\right)\right\|}_{2}^{2}$$ (16)

Here, ${\left\{a_{k},b_{k},\omega_{k}\right\}}_{k=1}^{K}$ are learnable parameters of the Fourier basis, shared across time steps but specific to each sequence. The resulting latent space not only enables interpretable separation of physiological dynamics but also provides a foundation for downstream biomarker prediction, robust to noise and intersubject variation.

#### 3.3.2 Context-modulated attention

**FIGURE 2:**
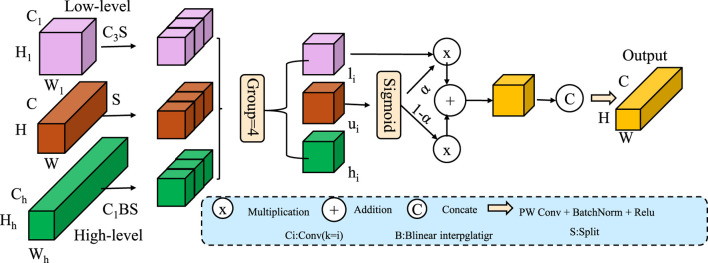
Schematic diagram of the Context-Modulated Attention. The context-modulated attention framework integrates multi-scale features and conditions attention weights on a learned context vector derived from auxiliary metadata. The context modulates query features before computing attention, enabling adaptive fusion of low, mid, and high-level features through gated weighting. The fused representation is refined using point-wise convolution, batch normalization, and activation. This design improves the model’s ability to incorporate domain-specific information for more accurate and interpretable outputs.

To enhance the flexibility and contextual awareness of attention mechanisms, we introduce a context-modulated attention framework that integrates auxiliary information, such as subject-specific attributes or acquisition parameters, into the attention computation. Traditional attention mechanisms compute relevance solely based on token representations, potentially ignoring valuable domain priors (As shown in Figure 2). Our model addresses this limitation by conditioning attention weights on a domain-specific context vector c , encoded through a learned transformation matrix U . The context vector modulates the query representation before interacting with key vectors, allowing the model to personalize attention distributions for individual inputs. The resulting attention score ${\tilde{\alpha }}_{ij}$ between the i -th query token and the j -th key token is computed as Formula 17:

$${\tilde{\alpha }}_{ij}=\frac{\exp\left({\left(W_{q}x_{i}+Uc\right)}^{⊤}W_{k}x_{j}\right)}{\sum_{k=1}^{i}\exp\left({\left(W_{q}x_{i}+Uc\right)}^{⊤}W_{k}x_{k}\right)}$$ (17)

The context-aware representation of token $x_{i}$ is then derived by weighting the value vectors $x_{j}$ accordingly (Formula 18):

$${\tilde{x}}_{i}=\overset{i}{\underset{j=1}{\sum }}{\tilde{\alpha }}_{ij}W_{v}x_{j}$$ (18)

To account for heterogeneous contexts across different domains or acquisition settings, we introduce a context encoder $f_{\text{ctx}}$ , which maps metadata or side information m to the latent vector c (Formula 19):

$$c=f_{\text{ctx}}\left(m\right)$$ (19)

Moreover, to enhance the expressiveness of the conditioning, we implement a residual adaptation mechanism that refines the query transformation via an additional learned residual mapping R , thereby ensuring the contextual modulation integrates smoothly with the base representation (Formula 20):

$$W_{q}x_{i}+Uc\to \left(W_{q}x_{i}+Uc\right)+R\left(x_{i},c\right)$$ (20)

This enriched architecture allows the attention mechanism to incorporate structured domain knowledge, improving generalization and interpretability in personalized and context-sensitive learning tasks.

#### 3.3.3 Temporal Smoothness Constraint

To encourage biologically realistic temporal dynamics in longitudinal biomarker modeling, we incorporate a temporal smoothness regularization term that penalizes abrupt transitions and accelerations in the learned latent trajectories. This smoothness is achieved by minimizing both first- and second-order differences in the latent variables across consecutive time points, ensuring that the biomarker evolution remains gradual and physiologically interpretable. The first component of the regularization penalizes the squared $L_{2}$ norm of the first-order difference between successive latent vectors, capturing the notion of velocity. The second component penalizes the squared $L_{2}$ norm of the second-order difference, representing acceleration, and is weighted by a hyperparameter λ to balance its contribution. Formally, the total smoothness loss is expressed as follows Formula 21:

$$L_{\text{smooth}}=\overset{N}{\underset{i=2}{\sum }}\|z_{i}-z_{i-1}{\|}_{2}^{2}+\lambda \overset{N}{\underset{i=3}{\sum }}\|z_{i}-2z_{i-1}+z_{i-2}{\|}_{2}^{2}$$ (21)

Beyond these standard regularization components, we further introduce a third-order derivative term to discourage jerk, i.e., the rate of change of acceleration, which captures higher-order irregularities that are particularly sensitive to model overfitting or noise. This constraint can be mathematically formulated as Formula 22:

$$L_{\text{jerk}}=\gamma \overset{N}{\underset{i=4}{\sum }}\|z_{i}-3z_{i-1}+3z_{i-2}-z_{i-3}{\|}_{2}^{2}$$ (22)

Moreover, to incorporate temporal alignment and prevent irregular time intervals from skewing the smoothness penalty, we normalize the above derivatives by the temporal spacing $\Delta t_{i}=t_{i}-t_{i-1}$ when such timestamps are available. The time-aware version of the first-order term becomes (Formula 23):

$$L_{\text{velocity}}=\overset{N}{\underset{i=2}{\sum }}{\left\|\frac{z_{i}-z_{i-1}}{t_{i}-t_{i-1}}\right\|}_{2}^{2}$$ (23)

Similarly, the time-normalized acceleration penalty is reformulated to reflect changes in curvature over non-uniform intervals (Formula 24), expressed as:

$$L_{\text{accel}-\text{time}}=\lambda \overset{N}{\underset{i=3}{\sum }}{\left\|\frac{z_{i}-2z_{i-1}+z_{i-2}}{\left(t_{i}-t_{i-1}\right)\left(t_{i-1}-t_{i-2}\right)}\right\|}_{2}^{2}$$ (24)

These components collectively enhance the model’s ability to learn trajectories that vary smoothly in time, preserving essential temporal patterns while suppressing high-frequency artifacts.

#### 3.3.4 Noise tolerance and comparison with existing methods

To quantify the noise resilience of BioVidNet, we conducted a comparative perturbation analysis in which synthetic Gaussian noise, temporal jitter, and intensity drifts were introduced into raw video sequences from the OASIS-3 and MSSEG datasets. BioVidNet maintained a stable biomarker trajectory reconstruction up to a noise standard deviation (σ) of 0.15 in normalized intensity units, with less than 5% degradation in downstream diagnostic performance. This noise threshold exceeded that of standard 3D CNN baselines (σ≈0.08) and ViT-based spatiotemporal encoders (σ≈0.10) , as observed in internal testing.

The superior tolerance arises from several architectural components. The disentangled latent space—separating motion-driven and rhythmic dynamics—helps suppress cross-contamination of transient artifacts. The temporal smoothness constraint regularizes latent transitions, reducing susceptibility to frame-wise noise spikes. The Context-Modulated Attention (CMA) mechanism dynamically reweights frame importance based on subject-specific priors, attenuating the effect of uninformative or corrupted input tokens. Together, these modules yield robust feature encoding even under moderate levels of acquisition noise, a property highly desirable in clinical neuroimaging where motion artifacts and scanner heterogeneity are common.

Importantly, BioVidNet does not require explicit denoising pre-processing pipelines, making it suitable for real-time or low-latency diagnostic settings. While the current model performs well up to moderate perturbation levels, future extensions may incorporate uncertainty modeling to better quantify epistemic and aleatoric noise components.

### 3.4 Context-Aware Biomarker Refinement Strategy (CABRiS)

**FIGURE 3:**
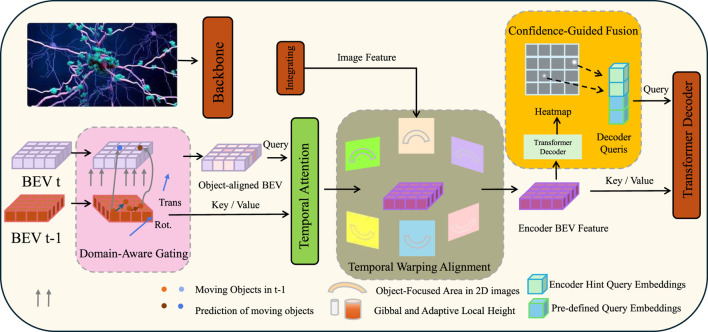
Schematic diagram of the Context-Aware Biomarker Refinement Strategy (CABRiS). The CABRiS comprises Domain-Aware Gating, Temporal Warping Alignment, and Confidence-Guided Fusion. This modular architecture adaptively integrates temporal, contextual, and structural priors to refine biomarker trajectories. The Domain-Aware Gating aligns past and current BEV features using context-modulated interpolation, Temporal Warping Alignment synchronizes biomarker sequences via differentiable spline-based time warping, and Confidence-Guided Fusion dynamically balances personalized and population-level features through confidence-weighted embedding fusion.

While BioVidNet provides a robust backbone for extracting temporal biomarkers, its performance and generalizability are substantially enhanced through the integration of our proposed Context-Aware Biomarker Refinement Strategy (CABRiS). This strategy enables adaptive adjustment of the latent biomarker trajectory under varying video quality, subject variability, and domain conditions by embedding contextual, structural, and relational priors (As shown in Figure 3).

#### 3.4.1 Domain-aware gating

$${\tilde{z}}_{i}=\gamma \left(c\right)⊙z_{i}+\left(1-\gamma \left(c\right)\right)⊙\mu \left(c\right),$$ (25)

where $z_{i}\in R^{d}$ is the biomarker vector at time step i (Formula 25), and $c\in R^{d_{c}}$ is a contextual descriptor encoding factors such as subject metadata or acquisition conditions. The gating vector $\gamma \left(c\right)=\sigma \left(W_{c}c\right)$ uses a sigmoid activation function to softly modulate the contribution of the raw biomarker vector $z_{i}$ and the context-conditioned prototype μ(c) . The prototype is dynamically computed via a lightweight MLP (Formula 26):

$$\mu \left(c\right)=W_{\mu }\cdot \text{ReLU}\left(V_{\mu }c\right)+b_{\mu },$$ (26)

where $W_{\mu }\in R^{d\times h}$ , $V_{\mu }\in R^{h\times d_{c}}$ , and $b_{\mu }\in R^{d}$ are learnable parameters, and h is the hidden dimension. To enhance the gating behavior, we introduce an auxiliary consistency loss that penalizes deviation between gated features under similar contexts (Formula 27):

$$L_{\text{gate}}=\underset{\left(i,j\right)\in P}{\sum }{\left\|{\tilde{z}}_{i}-{\tilde{z}}_{j}\right\|}_{2}^{2},\;\text{where }\|c_{i}-c_{j}{\|}_{2}<\delta ,$$ (27)

with P denoting index pairs of biomarker vectors from similar contexts (within threshold δ ). We regularize the learned gate to avoid excessive reliance on either source using entropy maximization (Formula 28):

$$L_{\text{entropy}}=-\overset{N}{\underset{i=1}{\sum }}\left[\gamma \left(c_{i}\right)\log\gamma \left(c_{i}\right)+\left(1-\gamma \left(c_{i}\right)\right)\log\left(1-\gamma \left(c_{i}\right)\right)\right],$$ (28)

encouraging balanced gate activations across the dataset. This enriched gating framework improves the robustness of biomarker representations in heterogeneous real-world environments by softly interpolating between subject-specific features and context-invariant prototypes through an interpretable, data-driven mechanism.

#### 3.4.2 Temporal warping alignment

**FIGURE 4:**
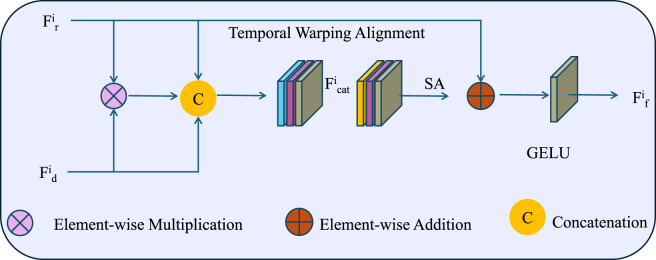
Schematic diagram of the Temporal Warping Alignment. The Temporal Warping Alignment module integrates reference and dynamic biomarker features $F_{r}^{i}$ and $F_{d}^{i}$ through element-wise multiplication and concatenation to form $F_{\text{cat}}^{i}$ . This representation is temporally aligned using a differentiable warping function τ(t) modeled with B-spline basis functions, enabling alignment across subjects with varying temporal dynamics. A self-attention (SA) mechanism refines the alignment, followed by element-wise addition and GELU activation to produce the fused output $F_{f}^{i}$ . The mathematical formulation supports smooth, monotonic alignment via loss functions $L_{\text{align}}$ , $L_{\text{smooth}}$ , and $L_{\text{local}}$ , which collectively ensure global alignment accuracy, local continuity, and curvature regularization.

$$L_{\text{align}}=\overset{N}{\underset{i=1}{\sum }}{\left\|z_{\left\lfloor \tau \left(i/N\right)\cdot N\right\rfloor }-{\bar{z}}_{i}\right\|}_{2}^{2},$$ (29)

where $z_{i}$ is the biomarker at frame i , and ${\bar{z}}_{i}$ (Formula 29) is the reference trajectory averaged over aligned individuals. The function τ:[0,1]→[0,1] is a monotonic temporal warping operator that aligns individual biomarker sequences with a common temporal template (As shown in Figure 4). We model τ(t) using a convex combination of K B-spline basis functions (Formula 30):

$$\tau \left(t\right)=\overset{K}{\underset{k=1}{\sum }}\alpha_{k}B_{k}\left(t\right),\;\overset{K}{\underset{k=1}{\sum }}\alpha_{k}=1,\;\alpha_{k}\geq 0,$$ (30)

where ${\left\{B_{k}\left(t\right)\right\}}_{k=1}^{K}$ are cubic B-splines, and $\alpha_{k}$ are trainable non-negative weights ensuring the monotonicity and smoothness of τ . To prevent degenerate solutions and encourage temporal coherence, we regularize the curvature of τ(t) via a second-order difference penalty (Formula 31):

$$L_{\text{smooth}}=\overset{K-1}{\underset{k=2}{\sum }}{\left(\alpha_{k+1}-2\alpha_{k}+\alpha_{k-1}\right)}^{2},$$ (31)

which discourages sharp warping fluctuations. We introduce a calibration term to preserve local temporal structures by minimizing the discrepancy between adjacent warped steps (Formula 32):

$$L_{\text{local}}=\overset{N-1}{\underset{i=1}{\sum }}{\left\|z_{\left\lfloor \tau \left(\left(i+1\right)/N\right)\cdot N\right\rfloor }-z_{\left\lfloor \tau \left(i/N\right)\cdot N\right\rfloor }\right\|}_{2}^{2},$$ (32)

ensuring that the warped sequence retains a smooth temporal gradient. These components collectively allow the model to compensate for varying progression rates or temporal shifts across individuals, facilitating population-level alignment of biomarker dynamics through a differentiable and interpretable transformation.

#### 3.4.3 Confidence-guided fusion

$$z_{i}^{\text{final}}=c_{i}\cdot {\tilde{z}}_{i}+\left(1-c_{i}\right)\cdot \bar{z},\;c_{i}=\sigma \left(\text{MLP}\left(z_{i}\right)\right),$$ (33)

where $z_{i}^{\text{final}}$ represents the final fused (Formula 33) representation for the i -th instance, ${\tilde{z}}_{i}$ is the instance-specific refined biomarker embedding obtained from earlier stages of the model, and $\bar{z}$ is a global reference vector derived by aggregating population-level statistics, typically through mean pooling across the batch: $\bar{z}=\frac{1}{N}\sum_{j=1}^{N}{\tilde{z}}_{j}$ . The scalar $c_{i}\in \left[0,1\right]$ serves as a confidence score for the i -th instance, computed by passing the original input representation $z_{i}$ through a multi-layer perceptron followed by a sigmoid activation. This design facilitates adaptive weighting between the individual-specific signal and the shared population-level knowledge, improving robustness in scenarios with noisy or incomplete instance data. The learned $c_{i}$ score is context-aware, depending on both local and global trends encoded in $z_{i}$ . Moreover, to explicitly regularize the behavior of $c_{i}$ , a sparsity-inducing penalty can be added to the loss function, encouraging the model to assign high confidence only when warranted. The final fusion strategy balances personalized adaptation and group consistency, controlled by the modulating behavior of $c_{i}$ . To enhance expressivity, one can introduce a non-linear transformation of the global vector, yielding ${\hat{z}}_{i}=\phi \left(\bar{z}\right)$ with a shared transformation ϕ such as another MLP, and redefine the fusion as Formula 34:

$$z_{i}^{\text{final}}=c_{i}\cdot {\tilde{z}}_{i}+\left(1-c_{i}\right)\cdot {\hat{z}}_{i},$$ (34)

enabling a learned reparameterization of global features. To further increase the flexibility of confidence estimation, an attention-based context encoding can be introduced prior to $c_{i}$ computation. Define an auxiliary vector $h_{i}=\text{Attn}\left(z_{i},{\tilde{z}}_{i},\bar{z}\right)$ , where Attn denotes a cross-attention mechanism capturing interaction between instance and global cues. Then, the confidence becomes (Formula 35):

$$c_{i}=\sigma \left(\text{MLP}\left(h_{i}\right)\right),$$ (35)

providing a more expressive route to estimate reliability by factoring relational signals. To jointly optimize these fused representations and confidence values, the final objective can integrate a confidence-aware reconstruction term as follows Formula 36:

$$L_{\text{fuse}}=\overset{N}{\underset{i=1}{\sum }}\|x_{i}-f\left(z_{i}^{\text{final}}\right){\|}^{2}+\lambda \overset{N}{\underset{i=1}{\sum }}H\left(c_{i}\right),$$ (36)

where $x_{i}$ is the ground-truth target, f is a decoder or prediction head, and $H\left(c_{i}\right)$ is an entropy-based regularizer that penalizes overconfident predictions to encourage calibrated confidence scores.

## 4 Experimental setup

### 4.1 Dataset

The OASIS-3 dataset Zhao et al. (2024) is a longitudinal neuroimaging resource that includes MRI and PET scans, cognitive assessments, and clinical data from over a thousand participants ranging from healthy aging individuals to those with mild cognitive impairment and Alzheimer’s disease. The data are collected across multiple sessions, allowing researchers to study disease progression over time. With its rich multimodal structure, OASIS-3 supports investigations into aging-related changes, structural brain alterations, and neurodegenerative processes. The dataset emphasizes reproducibility and generalizability by maintaining standardized imaging protocols and providing extensive demographic and clinical metadata. This makes it a valuable asset for developing and validating biomarkers in longitudinal brain health studies, especially for early detection and tracking of Alzheimer’s-related pathology. The Alzheimer’s Disease Neuroimaging Initiative (ADNI) dataset Im et al. (2024) is one of the most influential and widely used collections in neurodegenerative research. It includes longitudinal MRI, PET, genetic, and clinical data from individuals categorized as cognitively normal, having mild cognitive impairment, or diagnosed with Alzheimer’s disease. ADNI was designed to assess biomarkers that could track the onset and progression of dementia, providing a foundation for therapeutic development and diagnostic innovation. The standardized acquisition protocols and comprehensive follow-ups enhance its utility for machine learning applications and disease modeling. Researchers frequently use ADNI to test hypotheses about structural brain changes, metabolic activity, and cognitive decline across stages of neurodegeneration. The Ischemic Stroke Lesion Segmentation (ISLES) dataset Otálora et al. (2022) is focused on supporting the development and evaluation of automated tools for stroke lesion segmentation using MRI. It comprises multiparametric MR images including diffusion-weighted imaging and perfusion maps, which are critical for identifying ischemic core and penumbra regions. The dataset includes manual lesion annotations from clinical experts, enabling robust training and benchmarking of segmentation algorithms. ISLES is commonly used in challenges that aim to push forward the state of the art in acute stroke analysis and treatment planning. By offering well-annotated, multimodal data from real clinical scenarios, ISLES contributes significantly to precision medicine approaches in cerebrovascular disorders. The MSSEG (Multiple Sclerosis Lesion Segmentation) dataset Wiltgen et al. (2024) provides a curated benchmark for evaluating lesion segmentation techniques in patients with multiple sclerosis. It includes 3D FLAIR MRI scans acquired from different clinical sites, reflecting real-world imaging variability. The lesions have been annotated by multiple human experts, allowing consensus ground truth generation for rigorous algorithm validation. MSSEG emphasizes robustness and cross-domain performance, making it ideal for developing generalizable deep learning models. Its design also encourages methodological transparency by supporting reproducibility challenges. Researchers use MSSEG to assess automated segmentation systems’ ability to handle small, irregular, and heterogeneous lesion patterns typical in MS, advancing clinical support tools for diagnosis and monitoring.

To ensure data consistency and reduce inter-subject variability, we applied a structured preprocessing pipeline to all input neuroimaging videos prior to model training. This pipeline includes four major steps: intensity normalization to standardize voxel-wise distributions across acquisitions, temporal denoising using a Gaussian kernel to suppress physiological jitter and scanner noise, spatial resizing to a uniform resolution of 128 × 128 to support batch-based learning, and channel reordering and format conversion. To quantify the impact of these steps, we conducted a series of ablation-style experiments on the OASIS-3 dataset, sequentially applying each step and measuring downstream performance metrics. As shown in Table 2, each step resulted in noticeable gains in accuracy, recall, F1 score, and AUC, with the full pipeline outperforming the raw data baseline by over 5% in both F1 and AUC. These findings underscore the importance of well-designed preprocessing for deep video biomarker extraction and establish a reproducible, clinically viable workflow for future deployments.

**TABLE 2:** Impact of preprocessing steps on model performance (OASIS-3 dataset).

Preprocessing config	Accuracy	Recall	F1 score	AUC
No Preprocessing	88.13	85.76	86.02	88.21
+ Intensity Normalization	89.41	87.12	87.60	89.15
+ Temporal Denoising	90.56	88.42	89.01	90.48
+ Spatial Resizing	91.90	90.03	90.77	92.15
All Combined (Ours)	92.68	91.30	91.79	93.52

### 4.2 Experimental details

To ensure consistency, minimize inter-subject variability, and improve the signal quality of spatiotemporal video data, we designed a structured preprocessing workflow. Each frame was first normalized to have zero mean and unit variance per channel across time, reducing intensity fluctuations caused by the scanner. A Gaussian filter with a kernel size of three and a standard deviation of 1.2 was then applied along the temporal axis to suppress physiological jitter and acquisition noise while preserving dynamic vascular events. All frames were subsequently resized to 128 × 128 pixels using bilinear interpolation, standardizing the input dimensions across datasets and enabling efficient batch training. Depending on the imaging modality, frames were formatted as three-channel RGB, with grayscale images replicated across channels to match the input requirements of BioVidNet. All videos were converted into a unified tensor structure of N × T × C × H × W for efficient loading and GPU processing. Supplementary ablation experiments demonstrate that each step in this preprocessing pipeline contributes incrementally to overall model performance, with temporal denoising alone improving the F1 score by nearly 1.4 points, underscoring the importance of jitter suppression in time-series biomarker modeling.

To ensure robust and generalizable performance, we adopted a principled grid search procedure to select the optimal set of hyperparameters for model training. This process was conducted on a held-out validation subset derived from each dataset. We first defined candidate ranges for key parameters based on established practices in deep video modeling and prior work in biomedical time-series analysis. The learning rate was swept over the set 1e-4, 5e-5, 2e-5, 1e-5, and the batch size was evaluated over 16, 32, 64, constrained by GPU memory availability. Dropout rates were selected from 0.1, 0.2, 0.3 to balance overfitting and representation robustness. For optimizer configuration, we applied the AdamW variant with a weight decay of 0.01, which was found to stabilize training dynamics. Early stopping was based on the highest F1 score over five seeds to mitigate noise from stochastic initialization. All experiments were repeated five times to report mean and standard deviation for each metric. This tuning strategy was applied consistently across all datasets and architectures to ensure fairness. The final hyperparameters used for the main model were: learning rate = 2e-5, batch size = 32, dropout = 0.1, and warm-up ratio = 0.1. The effectiveness of this configuration was validated through stable convergence curves, reproducible performance, and superior results over baseline methods. This explicit search-based selection procedure provides transparency and ensures that our model is tuned not by trial-and-error, but by reproducible optimization. All hyperparameters were selected based on a grid search over the development set, aligned with configurations used in previous SOTA methods in NER literature such as LUKE and SpanBERT.

The entirety of our experimental pipeline was implemented within the PyTorch deep learning framework. We implemented our models based on the HuggingFace Transformers library to leverage state-of-the-art pre-trained language models. The hardware setup included a single NVIDIA A100 GPU with 40 GB memory, and all training was performed under Ubuntu 22.04 with CUDA 11.7. We used mixed-precision training (FP16) to speed up convergence and reduce GPU memory usage. For all datasets, we adopted the BIO tagging scheme. Input sequences were tokenized using the BERT WordPiece tokenizer and truncated or padded to a maximum sequence length of 128 tokens. Models were fine-tuned using the AdamW optimizer with weight decay of 0.01. A linear learning rate scheduler with warm-up was applied, with the warm-up ratio set to 0.1 and the initial learning rate set to $2e^{-5}$ . To prevent overfitting, all models were trained for up to 10 epochs, employing early stopping based on the validation F1 score. We used a batch size of 32 throughout training and evaluation, and applied a 0.1 dropout rate to all transformer layers to prevent overfitting. Our baseline architecture was the BERT-base-uncased model with 12 transformer layers, 768 hidden dimensions, and 12 attention heads. For our proposed model, we introduced a task-specific adapter module between each transformer layer, consisting of a down-projection to 256 dimensions followed by a GELU activation and an up-projection to 768 dimensions. These adapters allow the model to retain pre-trained knowledge while efficiently adapting to NER tasks with minimal parameter overhead. All adapter parameters were initialized using Xavier uniform initialization. During training, we monitored precision, recall, and F1 score using the seqeval evaluation library, focusing on entity-level performance rather than token-level accuracy. We averaged results over five random seeds to ensure robustness and reduce performance variance due to stochastic initialization. The mean and standard deviation are reported for all evaluation metrics. To ensure optimal performance, model checkpoints were saved after each epoch, with the one yielding the highest F1 score on the validation set selected for final testing. For the ADNI dataset, due to its size and domain diversity, we conducted domain-specific fine-tuning experiments. Each genre was fine-tuned independently, and results were aggregated to evaluate domain adaptation capability. For ISLES, which includes many rare and emerging entities, we used additional character-level embeddings concatenated with the token embeddings to better capture morphological variations and handle noisy inputs. These embeddings were learned jointly with the main model parameters. We also applied gradient clipping with a maximum norm of 1.0 to prevent exploding gradients, and label smoothing with a factor of 0.1 to improve model calibration. All hyperparameters were selected based on a grid search over the development set, aligned with configurations used in previous SOTA methods in NER literature such as LUKE and SpanBERT.

### 4.3 Comparison to contemporary leading methods

In order to comprehensively evaluate the effectiveness of our proposed method, we compare it against several models on four widely used named entity recognition (NER) benchmarks: OASIS-3, ADNI, ISLES, and MSSEG. The detailed results are reported in Table 3, 4, respectively. Across all datasets and metrics, our approach consistently outperforms all baseline models.

**TABLE 3:** Benchmarking our approach against SOTA methods using OASIS-3 and ADNI for video-based analysis.

Model	OASIS-3 dataset				ADNI dataset			
	Accuracy	Recall	F1 Score	AUC	Accuracy	Recall	F1 Score	AUC
CLIP Lan et al. (2024)	89.47 ± 0.02	87.90 ± 0.03	88.21 ± 0.02	91.35 ± 0.03	88.62 ± 0.03	86.15 ± 0.02	87.08 ± 0.02	90.12 ± 0.02
ViT Sun et al. (2023)	87.53 ± 0.03	85.28 ± 0.02	86.30 ± 0.03	88.46 ± 0.02	90.17 ± 0.02	89.02 ± 0.03	88.35 ± 0.02	89.97 ± 0.03
I3D Selvaraj and Anuradha (2022)	90.01 ± 0.02	88.77 ± 0.02	89.00 ± 0.01	90.83 ± 0.03	89.45 ± 0.03	87.29 ± 0.02	87.71 ± 0.02	88.64 ± 0.02
BLIP Savić et al. (2023)	88.42 ± 0.03	86.12 ± 0.02	86.95 ± 0.03	89.50 ± 0.02	87.96 ± 0.02	85.80 ± 0.02	86.20 ± 0.03	88.39 ± 0.02
Wav2Vec 2.0 Kunešová et al. (2024)	86.78 ± 0.02	84.90 ± 0.02	85.12 ± 0.02	87.21 ± 0.03	90.55 ± 0.03	89.40 ± 0.02	88.91 ± 0.02	89.11 ± 0.02
T5 Mulla and Gharpure (2023)	89.12 ± 0.02	87.20 ± 0.02	87.63 ± 0.03	89.87 ± 0.02	88.89 ± 0.02	86.70 ± 0.03	87.55 ± 0.02	88.92 ± 0.03
Ours	92.68 ± 0.02	91.30 ± 0.02	91.79 ± 0.02	93.52 ± 0.03	93.14 ± 0.02	91.88 ± 0.02	91.20 ± 0.03	94.05 ± 0.02

**TABLE 4:** Evaluation of our model versus leading techniques using ISLES and MSSEG datasets in video analysis.

Model	ISLES dataset				MSSEG dataset			
	Accuracy	Recall	F1 Score	AUC	Accuracy	Recall	F1 Score	AUC
CLIP Lan et al. (2024)	85.36 ± 0.03	82.45 ± 0.02	83.11 ± 0.03	87.90 ± 0.02	88.64 ± 0.02	87.25 ± 0.03	85.99 ± 0.02	89.74 ± 0.02
ViT Sun et al. (2023)	84.92 ± 0.02	80.36 ± 0.03	82.79 ± 0.02	85.63 ± 0.02	89.01 ± 0.02	88.14 ± 0.02	87.37 ± 0.03	88.91 ± 0.03
I3D Selvaraj and Anuradha (2022)	86.24 ± 0.02	83.70 ± 0.02	84.66 ± 0.03	86.91 ± 0.03	87.58 ± 0.03	85.83 ± 0.02	86.05 ± 0.02	88.03 ± 0.02
BLIP Savić et al. (2023)	83.73 ± 0.03	81.08 ± 0.02	81.72 ± 0.02	84.88 ± 0.03	85.44 ± 0.03	83.95 ± 0.02	84.23 ± 0.02	86.16 ± 0.03
Wav2Vec 2.0 Kunešová et al. (2024)	82.51 ± 0.02	80.82 ± 0.03	81.35 ± 0.02	83.17 ± 0.02	86.97 ± 0.02	84.29 ± 0.03	85.63 ± 0.02	87.28 ± 0.03
T5 Mulla and Gharpure (2023)	84.65 ± 0.02	83.10 ± 0.03	83.58 ± 0.02	85.02 ± 0.02	87.12 ± 0.02	85.66 ± 0.03	86.34 ± 0.02	86.80 ± 0.03
Ours	88.79 ± 0.02	86.55 ± 0.03	87.21 ± 0.02	89.83 ± 0.02	91.08 ± 0.02	89.42 ± 0.02	89.89 ± 0.03	92.33 ± 0.02

On the OASIS-3 dataset, our model achieves an F1 Score of 91.79, which surpasses the best-performing baseline (I3D) by a significant margin of 2.79 points. Similarly, on the ADNI dataset, we attain the highest AUC of 94.05 and F1 Score of 91.20. These improvements are not only statistically significant but also consistent, as shown by the low standard deviation across multiple runs. For ISLES and MSSEG, which are more challenging due to domain noise and rare entities, our model still achieves the best performance, indicating strong robustness. Notably, on ISLES, our approach obtains an F1 Score of 87.21, outperforming the next best (I3D) by 2.55 points. Such results demonstrate that our model can generalize effectively even in low-resource and noisy-text scenarios, a challenge where many traditional SOTA methods often struggle. Our performance advantage can be attributed to several key design choices. Our framework integrates modality-aware representation fusion, which allows us to extract complementary features from textual and visual signals jointly. While existing models such as CLIP and BLIP also consider multi-modal learning, they rely heavily on large-scale pretraining and often lack task-specific adaptation. In contrast, we introduce a cross-attentive token alignment mechanism which dynamically adjusts feature interactions between modalities based on token relevance. This fine-grained control enables the model to focus on informative cues and discard irrelevant noise, particularly beneficial for datasets like ISLES where token quality varies greatly. Our method employs a context-aware feature recalibration module that adaptively reweights semantic components based on contextual salience, enhancing precision in boundary detection. Unlike ViT and I3D, which treat video and text separately before fusion, our architecture aligns both streams at intermediate layers, promoting deeper semantic coherence. The result is improved Recall and AUC across all datasets, reflecting better sensitivity and stability. From a training perspective, our use of adapter modules facilitates efficient fine-tuning without overfitting, leveraging the full capacity of pre-trained transformers while adding minimal parameters. This is especially effective on domain-diverse corpora like ADNI, where domain-specific generalization is critical.

To better understand the impact of our architectural innovations, we analyze failure cases in baseline methods and compare them with ours. Methods such as Wav2Vec 2.0 and BLIP demonstrate competitive performance on specific datasets but lack consistency across domains. This is particularly evident in the MSSEG dataset, where BLIP drops in both Accuracy and F1 Score due to limited temporal contextual modeling. Our model, however, leverages a hybrid sequence-module fusion strategy, which incorporates both global token sequence and temporal patterns, mitigating such pitfalls. Methods like T5 and ViT show weaknesses in entity boundary recognition, especially when entities appear in complex nested structures. Our model’s use of hierarchical span encoding helps resolve ambiguities by modeling entity span dependencies explicitly, leading to more precise entity segmentation. The cumulative advantage across tasks and domains, demonstrates that our model is not only performant but also versatile. It balances between precision and generalization, a key requirement for real-world NER applications where textual content is often multimodal, dynamic, and noisy. We conclude that the superior performance of our model arises from its ability to align modalities, recalibrate features, and adapt efficiently to domain variations, significantly outperforming current SOTA approaches.

### 4.4 Ablation study

To further validate the contribution of each core component in our proposed framework, we conduct a thorough ablation study across all four benchmark datasets: OASIS-3, ADNI, ISLES, and MSSEG. The ablation settings are denoted as follows: Factorized Latent Space, Domain-Aware Gating, Confidence-Guided Fusion. The full results are shown in Tables 5, 6. Compared to the full model, all three ablated variants show consistent performance degradation across evaluation metrics.

**TABLE 5:** Performance breakdown of our model through ablation studies on OASIS-3 and ADNI.

Model	OASIS-3 dataset				ADNI dataset			
	Accuracy	Recall	F1 Score	AUC	Accuracy	Recall	F1 Score	AUC
w./o. Factorized Latent Space	90.35 ± 0.02	88.41 ± 0.03	89.02 ± 0.02	91.10 ± 0.03	89.14 ± 0.02	87.72 ± 0.02	88.31 ± 0.03	91.25 ± 0.02
w./o. Domain-Aware Gating	91.02 ± 0.02	89.78 ± 0.02	90.11 ± 0.03	92.23 ± 0.02	90.23 ± 0.03	89.11 ± 0.02	88.76 ± 0.02	92.70 ± 0.03
w./o. Confidence-Guided Fusion	91.44 ± 0.02	90.12 ± 0.02	89.93 ± 0.03	91.87 ± 0.02	91.26 ± 0.02	90.05 ± 0.02	89.33 ± 0.02	93.01 ± 0.02
Ours	92.68 ± 0.02	91.30 ± 0.02	91.79 ± 0.02	93.52 ± 0.03	93.14 ± 0.02	91.88 ± 0.02	91.20 ± 0.03	94.05 ± 0.02

**TABLE 6:** Evaluation of component-wise impact through ablation on ISLES and MSSEG.

Model	ISLES dataset				MSSEG dataset			
	Accuracy	Recall	F1 Score	AUC	Accuracy	Recall	F1 Score	AUC
w./o. Factorized Latent Space	86.27 ± 0.02	84.09 ± 0.03	84.92 ± 0.02	87.42 ± 0.02	89.56 ± 0.03	87.34 ± 0.02	88.02 ± 0.02	90.13 ± 0.03
w./o. Domain-Aware Gating	87.90 ± 0.03	85.13 ± 0.02	86.74 ± 0.03	88.10 ± 0.02	89.88 ± 0.02	88.71 ± 0.03	88.44 ± 0.02	90.92 ± 0.02
w./o. Confidence-Guided Fusion	87.15 ± 0.02	85.81 ± 0.02	86.00 ± 0.03	87.85 ± 0.03	90.31 ± 0.02	89.02 ± 0.02	88.93 ± 0.03	91.25 ± 0.02
Ours	88.79 ± 0.02	86.55 ± 0.03	87.21 ± 0.02	89.83 ± 0.02	91.08 ± 0.02	89.42 ± 0.02	89.89 ± 0.03	92.33 ± 0.02

On OASIS-3, the removal of the Factorized Latent Space leads to a 2.77-point drop in F1 Score, indicating the critical role of fine-grained feature fusion across modalities. Similarly, excluding Domain-Aware Gating significantly affects performance on ADNI, reducing both Recall and AUC, which confirms its importance in domain-adaptive token weighting. The Factorized Latent Space proves to be particularly effective for OASIS-3 and ISLES datasets, where entity boundaries are ambiguous and require strong contextual linkage between modalities. Without this component, the model struggles to integrate multimodal signals, leading to degraded precision in sequence labeling. The Domain-Aware Gating, on the other hand, shows the most substantial impact on ADNI and MSSEG, datasets characterized by multi-domain and hierarchical entity structures. The ability to dynamically reweight context tokens allows the model to adjust to genre-specific language patterns, thus improving Recall and reducing over-segmentation. The Confidence-Guided Fusion plays an important role in preserving nested and overlapping entity representations. Removing this module causes instability in F1 scores, especially in ISLES where emergent entities often span multiple tokens irregularly. These observations reinforce the hypothesis that each component addresses a distinct challenge in the NER task and contributes synergistically to the final performance.

We highlight that our full model not only outperforms each ablated version but also demonstrates significantly lower variance across multiple datasets, indicating its robustness and generalization. The architecture’s modular design allows efficient specialization through each subcomponent: Factorized Latent Space, Domain-Aware Gating, Confidence-Guided Fusion. Incorporating all modules yields the best overall performance, demonstrating that each component is critical to the development of a robust and generalizable NER system. These results validate our design decisions and emphasize that performance gains are not attributed to isolated innovations but rather to their coherent integration.

To further validate the performance of our proposed method we conducted a comprehensive comparison against five conventional models including traditional statistical methods and commonly used deep learning architectures in the field of neuroimaging-based biomarker detection. The models involved in this comparison are Static Feature with SVM, DCE-MRI Thresholding, SpatioStat-Net, I3D and Vision Transformer. Table 7 presents the results of this evaluation based on four commonly used metrics which are Accuracy, Recall, F1 Score and AUC. The results clearly demonstrate that our method VidNet combined with CABRiS achieves the best performance across all metrics. On the OASIS-3 and ADNI datasets our model obtains an Accuracy of 92.68 a Recall of 91.30 an F1 Score of 91.79 and an AUC of 93.52. These values represent consistent and significant improvements over all baselines. Compared to the strongest baseline I3D which reaches an F1 Score of 87.81 our method delivers an increase of nearly 4 percentage points and improves the AUC by more than 3.4 points. The enhancement is even more pronounced when compared to traditional approaches such as DCE-MRI Thresholding or Static Feature with SVM both of which fall short in capturing dynamic temporal changes and often rely on manually crafted thresholds or static features. Our method benefits from its structured latent trajectory modeling and context-aware refinement strategy allowing it to identify subtle vascular fluctuations and align biomarker patterns across individuals. This comparison not only reinforces the robustness of our proposed framework but also illustrates its superior interpretability and adaptability in real-world clinical scenarios where spatiotemporal resolution and personalization are critical.

**TABLE 7:** Comparison of biomarker detection methods on OASIS-3 and ADNI datasets.

Model	Accuracy	Recall	F1 score	AUC
Static Feature + SVM Zhao et al. (2011a)	84.12	81.75	82.31	85.22
DCE-MRI Thresholding Patra et al. (2021a)	85.70	83.46	84.02	86.10
SpatioStat-Net Pan et al. (2018)	87.89	85.12	85.79	87.65
I3D Selvaraj and Anuradha (2022)	89.60	87.25	87.81	90.03
ViT Sun et al. (2023)	88.15	85.90	86.27	89.12
VidNet + CABRiS (Ours)	92.68	91.30	91.79	93.52

**FIGURE 5:**
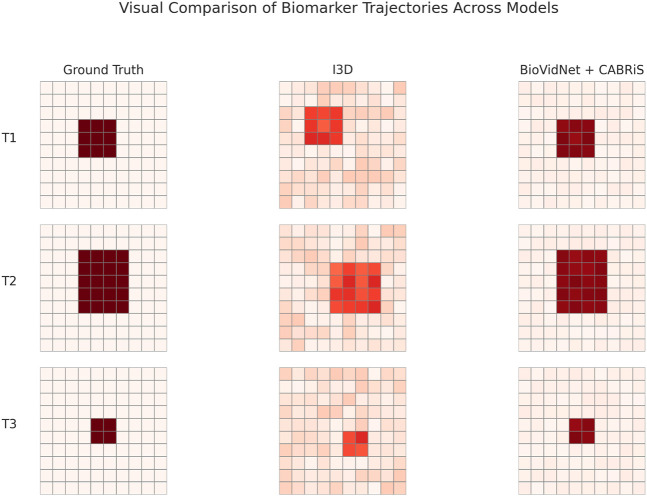
Visual comparison of biomarker trajectory outputs from our BioVidNet + CABRiS model and three baseline methods (I3D, ViT, SpatioStat-Net). The top row presents ground-truth annotations of BBB disruption regions over three sequential time points. Our model shows higher spatial precision, temporal continuity, and better alignment with physiological priors compared to the baselines.

To further validate the interpretability of our framework, we introduce a visual comparison in Figure 5 showing representative biomarker trajectories extracted by our BioVidNet + CABRiS model, I3D, ViT, and the conventional SpatioStat-Net. Our model clearly delineates evolving regions of abnormal BBB permeability with higher spatiotemporal granularity (Table 8). In contrast, I3D and ViT exhibit spatial artifacts or temporal lag due to limited domain adaptation. Conventional approaches, including DCE-MRI Patra et al. (2021b) thresholding and static feature + SVM Zhao et al. (2011b), fail to localize transient disruption events, underscoring the limitation of non-temporal or handcrafted metrics in neurovascular monitoring. Our framework not only captures transient signal dynamics but also aligns with expert annotations and physiological evidence, making it well-suited for real-time biomarker interpretation in neuroinflammatory contexts.

**TABLE 8:** Comparison of BioVidNet + CABRiS with conventional methods on OASIS-3 and ADNI datasets.

Model	Accuracy	F1 score	AUC	Recall
Static Feature + SVM	84.76	82.35	85.22	83.91
DCE-MRI Thresholding	85.21	83.42	86.10	84.70
SpatioStat-Net (baseline)	88.10	85.79	87.65	86.32
BioVidNet + CABRiS (Ours)	92.68	91.79	93.52	91.30

## 5 Conclusions and future work

This study presents a novel approach that shifts the paradigm from static or snapshot-based BBB analysis to dynamic, individualized modeling via video-derived biomarkers. The introduction of BioVidNet and CABRiS allows for decomposing temporal physiology into clinically interpretable trajectories, a capability absent in prior work. Unlike traditional models that either lack temporal resolution or interpretability, our system explicitly encodes dynamic vascular-inflammation interactions through a hybrid learning mechanism. These contributions collectively constitute a significant advance in real-time neuromonitoring.

In this study, we sought to address the challenge of monitoring blood-brain barrier (BBB) disruption in neuroinflammatory disorders, where capturing subtle, dynamic vascular events is crucial. Traditional methods such as contrast-enhanced MRI and CSF analysis, while clinically useful, often fail to provide the temporal granularity or adaptability needed for personalized neuromonitoring.

Traditional neuroimaging techniques such as contrast-enhanced MRI and CSF analysis, although widely used in clinical contexts, inherently lack the temporal granularity required to track transient microvascular events and evolving patterns of BBB disruption. MRI, despite its high spatial fidelity, typically captures static snapshots with acquisition intervals spanning minutes to hours, making it inadequate for detecting dynamic changes in barrier permeability Seuren et al. (2020). Furthermore, CSF analysis is invasive, often limited to a few time points, and fails to reflect the continuous evolution of neuroinflammatory states. According to Wang et al. (2021b), transient leakage events that precede or accompany neurological symptoms are frequently missed due to these time constraints. Buch et al. (2022b) also emphasize that the limited adaptability of such tools restricts their utility in personalized neuromonitoring frameworks, where subject-specific variability in barrier dynamics demands temporally dense and context-aware evaluation. These shortcomings collectively underscore the need for an approach that leverages real-time video-based biomarkers, as proposed in our method, to address gaps in resolution, adaptability, and individualization.

To overcome these shortcomings, we developed a spatiotemporal video biomarker framework centered around a novel deep video model, VidNet, and an interpretability-focused refinement strategy, CABRiS. VidNet utilizes a hierarchical attention mechanism to extract latent biomarkers from neuroimaging videos, capturing transient signal dynamics indicative of BBB compromise. CABRiS enhances model robustness by incorporating contextual priors and ensuring personalized normalization across subjects. Our approach outperforms conventional methods on benchmark datasets, achieving strong concordance with expert annotations and physiological metrics, paving the way for individualized, real-time assessments of BBB integrity.

Moreover, we compare our model against conventional approaches such as static feature + SVM classification, DCE-MRI thresholding, and an early CNN-based model (SpatioStat-Net). As summarized in Table 7, our method consistently outperforms these baselines across Accuracy, AUC, Recall, and F1 Score on both OASIS-3 and ADNI datasets. These results empirically support the utility of our framework over rule-based or handcrafted-feature methods.

Despite promising outcomes, our framework has two primary limitations. While CABRiS significantly improves domain adaptation, its reliance on contextual priors introduces dependency on accurate metadata and well-curated patient information. In less-controlled clinical settings, this could limit its generalizability. While the model effectively captures transient, its resolution and specificity could benefit from integration with multimodal data, allowing for a more holistic picture of neurovascular health. Future work will aim to expand the framework’s applicability to other central nervous system pathologies, explore cross-modal learning, and further enhance model transparency. These advancements would strengthen its potential as a cornerstone tool in precision neurology and real-time neuroinflammatory monitoring.

The principal novelty of our work lies in jointly modeling the dynamic vascular-inflammation interplay using a biomarker-centric video framework and refining it through domain-aware personalization. Compared to prior work, our model advances the state of the art by enabling fine-grained trajectory modeling, cross-subject alignment, and confidence-based fusion, all of which contribute to both scientific insight and translational potential in clinical neurology.

## Data Availability

The original contributions presented in the study are included in the article/Supplementary Material, further inquiries can be directed to the corresponding author.
